# Propagation of avian influenza virus in embryonated ostrich eggs

**DOI:** 10.4102/ojvr.v89i1.2011

**Published:** 2022-12-08

**Authors:** Agnes T. Laleye, Modupeore Adeyemi, Celia Abolnik

**Affiliations:** 1 Department of Production Animal Studies, Faculty of Veterinary Science, University of Pretoria, Pretoria, South Africa; 2 National Veterinary Research Institute, Vom, Nigeria

**Keywords:** avian influenza virus, propagation, ostrich, embryonated eggs, protocol

## Abstract

Influenza A viruses (IAVs) are typically isolated and cultured by successive passages using 9- to 11-day-old embryonated chicken eggs (ECEs) and in 14-day old ECEs for virus mutational studies. Real-time reverse transcription-polymerase chain reaction tests (RT-PCRs) are commonly used for IAV diagnosis, but virus isolation remains invaluable in terms of its high sensitivity, providing viable isolates for further studies and the ability to distinguish between viable and nonviable virus. Efforts at isolating ostrich-origin IAVs from RT-PCR positive specimens using ECEs have often been unsuccessful, raising the possibility of a species bottleneck, whereby ostrich-adapted IAVs may not readily infect and replicate in ECEs, yet the capacity of an ostrich embryo to support the replication of influenza viruses has not been previously demonstrated. This study describes an optimised method for H5 and H7 subtype IAV isolation and propagation in 28-day old embryonated ostrich eggs (EOEs), the biological equivalent of 14-day old ECEs. The viability of EOEs transported from breeding sites could be maximised by pre-incubating the eggs for 12 to 14 days prior to long-distance transportation. This method applied to studies for ostrich-adapted virus isolation and *in ovo* studies will enable better understanding of the virus-host interaction in ostriches and the emergence of potentially zoonotic diseases.

## Introduction

Avian influenza (AI) is a notifiable disease caused by influenza A viruses (IAVs) in the family *Orthomyxoviridae* (OIE 2017). Influenza A viruses have a wide host range, including birds and mammals, and are of zoonotic importance (OIE 2017). Two pathotypes of IAVs have been described – the severe, highly pathogenic avian influenza (HPAI) and low-pathogenicity AI (LPAI), which is associated with mild respiratory disease (Alexander 2000; OIE 2017).

The first outbreak of AI in ostriches, reported in the Oudtshoorn area in South Africa in the early 1980s, was caused by an H7N1 LPAI virus with a low pathogenicity index in chickens (Allwright et al. 1993). Subsequent infections of ostriches with LPAI viruses continued to be reported, including H1N2, H5N9 LPAI, H6N1, H6N8, H7N1 LPAI, H7N7 LPAI, H9N2 and H10N1 (Abolnik et al. 2016; Olivier 2006). Ostriches are also susceptible to infection with HPAI viruses, although such infections generally cause mild clinical signs (Abolnik et al. 2009, 2012, 2016; De Benedictis et al. 2007; Manvell et al. 2005). Interestingly, outbreaks of H7 LPAI among young flocks were observed to have presented with more severe clinical signs and higher mortalities in the ostriches than H5N2 HPAI outbreaks (Abolnik et al. 2009). Outbreaks of H5N2 HPAI in ostriches occurred in 2004 in the Eastern Cape province and in 2006 and 2011 in the Western Cape province of South Africa (Abolnik et al. 2012; Van Helden et al. 2016), with devastating economic impacts on the South African ostrich industry through control measures and trade restrictions. The H5N2 outbreaks were highly localised, with HPAI emerging from LPAI virus precursors in the ostriches (Abolnik 2007; Abolnik et al. 2012, 2016), compared with the outbreaks caused post-2017 by clade 2.3.4.4 H5Nx HPAI viruses introduced by wild migratory birds from the northern hemisphere (Abolnik et al. 2019).

Influenza A viruses are typically isolated and cultured by successive passages using 9- to 11-day-old embryonated chicken eggs (ECEs), which is considered the gold standard for virus detection by the OIE (Word Organization for Animal Health 2017; Spackman & Killian 2014). In recent times, sensitive nucleic acid detection assays like real-time reverse transcription-polymerase chain reaction tests (RT-PCRs) are more commonly used for diagnostic purposes, but virus isolation remains invaluable in terms of its high sensitivity, providing viable isolates for further studies and the ability to distinguish between viable and nonviable virus (Burrell, Howard & Murphy 2017).

Efforts at isolating ostrich-origin IAVs from RT-PCR positive specimens using ECEs have often been unsuccessful (Abolnik et al. 2016), for various possible reasons. For example, a recent study found that bacteria present in the ostrich trachea have antiviral effects on IAV, which may partially explain the failure to isolate IAVs from RT-PCR positive tracheal swabs (Abolnik et al. 2021). However, the possibility of a species bottleneck remains, whereby ostrich-adapted IAVs may not readily infect and replicate in chicken ECEs. If the latter is true, then ostrich-origin IAVs may replicate more readily in ostrich embryos without the need for prior adaptation. Previously, molecular markers arising from specific mutations in different segments of the IAV genome have been shown to be responsible for adaptation of avian viruses to mammalian hosts (Gabriel, Herwig & Klenk 2008; Hatta et al. 2001; Subbarao, London & Murphy 1993).

The average weight of an ostrich egg, at 1600 g, is around 25 times that of a chicken’s, and the shell thickness of an ostrich egg varies from 1.5 mm to 2.2 mm compared to the chicken egg shell, which has a thickness of 0.3 mm to 0.4 mm (Kokoszyński 2017). The embryonic development of ostriches is comparable to that of chickens; the incubation period of ostriches is exactly double that of chickens, and any particular stage of ostrich embryonic development can be obtained by reference to the corresponding developmental stage in the chicken embryo (Deeming 1997; Brand et al. 2017). Intensive ostrich farming started in the late 1800s (Shanawany 1995), and artificial incubation conditions for ostrich eggs have been well studied (Brand et al. 2017). The hatchability of artificially incubated ostrich eggs is normally between 50% and 60% of all the fertile eggs that were set (Van Schalkwyk et al. 2000), while that of chicken eggs ranges between 80% and 95%, depending on a number of variables including size, weight and duration of storage (Ayeni et al. 2020).

The transmission dynamics and pathogenesis of IAV in ostriches are relatively poorly understood in comparison to other poultry, because *in vivo* studies require high bio-containment animal facilities. Ostriches are large and potentially dangerous as adults, and birds of any age (but particularly chicks) are prone to stress and are difficult to manage indoors. Fourteen-day-old ECEs have been used with success as an alternative to live chickens to passage IAVs and study the emergence of HPAI from LPAI precursors (Laleye & Abolnik 2020; Seekings et al. 2020), but the growth of IAVs or any other viruses has not been explored in ostrich eggs before. This study aimed at developing and optimising a method for propagating IAVs in embryonated ostrich eggs (EOEs), as a possible substitute to the use of sentient ostriches for experimental purposes and as an alternative method to facilitate the recovery of viable ostrich-origin viruses in cases where other isolation methods are unsuccessful.

## Materials and methods

### Ostrich egg source and transport

Batches of fresh EOEs with an anticipated fertility rate of 50% – 60% (Van Schalkwyk et al. 2000) were purchased from Klein Karoo International (KKI) Research Laboratory in Oudtshoorn, South Africa. Eggs were sourced from ostrich farms that were certified AI-free by the Department of Agriculture, Forestry and Fisheries. These farms are tested monthly by serological methods to prove freedom of infection. In total, 102 ostrich eggs were used in the study.

The University of Pretoria, Faculty of Veterinary Science in Pretoria, Gauteng province, where the study was performed is located 1207 km from the KKI Research Laboratory, Western Cape province, and the closest airports for air freight are located in Johannesburg and George, respectively. The distance over which the EOEs had to be transported and still remain viable presented a significant challenge; therefore, different approaches for safe transport were investigated. In each case, the eggs were individually encased in several layers of plastic bubble wrap and placed into cardboard boxes filled with shredded paper for insulation. Boxes were clearly marked as ‘fragile’.

*Method 1*: Air freight with a commercial courier service. The package was collected and delivered door-to-door by the courier company.

*Method 2*: Road freight with meat trucks. The package was transported by a truck shipping packaged ostrich meat for international export purposes from Oudtshoorn to OR Tambo International airport in Johannesburg, where it was personally collected on arrival.

*Method 3*: Air transport as hand luggage. Personnel from the KKI laboratory travelled by air to OR Tambo International airport in Johannesburg, where the package was collected. The EOEs were first incubated at the KKI laboratory for 12 to 14 days prior to packaging and transport at recommended temperature and humidity in an incubation chamber.

### Egg handling and incubation

#### Egg storage

All experiments were conducted in the Poultry Biosafety Level 3 (BSL 3) facility at the Faculty of Veterinary Science. Upon receipt, eggs were left undisturbed for a period of 12 h, usually overnight, to allow them to stabilise. Thereafter, the eggs were candled using a portable hand-held LED egg candling lamp (Ecotao, South Africa) to determine the air-cell position, which was marked on the shell surface with a pencil or permanent marker. Unincubated eggs that were not used immediately were stored in padded plastic crates, placed in an isolation room in the BSL 3 facility that was separated from the area used for working with live virus. The room temperature was maintained at 18 °C with the air conditioning unit in accordance with the specified optimal temperature (15 °C to 20 °C) for the storage of fertile ostrich eggs (Swart, Rahn & De Kock 1987; Van Schalkwyk et al. 2000). To maintain the required relative humidity of 75% to 80%, bowls of water were placed in the room. The room temperature and relative humidity were monitored with a digital temp-hygrometer monitor (PMI, South Africa). The eggs were stored for a maximum of seven days before incubation, with the air-cell positioned horizontally and the eggs turned manually at least once a day.

#### Setting eggs in the incubator

Eggs stored at 18 °C were allowed to acclimatise for at least 12 h at room temperature (25 °C) before setting the incubator, to avoid an undesirable increase in humidity inside the incubator, which may potentially enhance growth and multiplication of microbes (Brand et al. 2014). An SH1700 model incubator (SureHatch, Brackenfell, South Africa) was used in this study. The SH1700 model incubator has an internal volume of 1.28 m^3^, digital temperature control, and an automatic egg turner that rotates through 90° every hour. The incubator was customised with crates for ostrich eggs, with a capacity for 30 eggs. Eggs were set with the air-cell up from day one.

Temperature and the relative humidity were set at 36 °C (± 0.5 °C) and 28%, respectively (Brand et al. 2014), with daily monitoring and recording on a log. Eggs were candled daily and those containing dead embryos were removed and discarded. After every incubation batch, the interior of the incubator was cleaned and disinfected with 10% F10 SC veterinary disinfectant solution.

### Viruses

Two ostrich-origin LPAIV originally isolated in ECEs, A/ostrich/South Africa/325863/2015 (H5N2) and A/ostrich/South Africa/ORD/2012 (H7N1), were used in the study. The viruses were propagated in specific pathogen-free ECEs (AviFarms Pty [Ltd], Pretoria, South Africa), and EID_50_ titres were determined according to the method of Reed and Muench (1938). At the time of the experiments, the stocks had been passaged three times in ECEs and the virus concentration given at 10^6^ 50% egg infectious doses (EID_50_)/0.1 mL, prescribed viral dose for challenge studies in avian species (Li et al. 2016).

### Avian influenza virus propagation in embryonated ostrich eggs

At the 28th day of incubation, viable EOEs were removed from the incubator and placed in the Biosafety cabinet for the inoculation process. The egg shell surface was disinfected with 70% ethanol and left for a few minutes to dry. A Dremel 4000 drill (Dremel, South Africa) was used to carefully drill an inoculating hole of 1 mm – 2 mm diameter in the egg shell. Using a 1 mL syringe with 21 gauge (G) x1” needle, 0.5 mL of allantoic fluid containing 10^6^ EID_50_/ 0.1 mL of virus was inoculated into the allantoic sac of 3 EOEs each by inserting the entire needle vertically through the inoculating hole. Inoculating holes were sealed with adhesive stickers.

Inoculated EOEs were incubated for 3–5 days per passage, under the same conditions as uninoculated eggs, but without turning, and were candled every 24 h. At the end of the incubation period, the eggs were chilled overnight at 4 °C. The allantoic fluid as well as embryonic tissues were collected and tested for haemagglutinating activity (HA) using 1% chicken red blood cells according to the standard procedure (OIE 2017), and bacteriological tests on blood agar (BA). Tissues collected from the embryos included trachea, heart, liver, spleen, lungs, intestine, kidneys and brain. The embryonic tissues were pooled and homogenised in antibiotic solution containing 50 mg gentamycin (Virbac) and 100 mg enrofloxacin (Baytril) per litre, using a Silent Crusher M Homogenizer (Heildoph Schwabach, Germany). The homogenates were clarified at 1000 × g and supernatant collected for HA and bacteriological tests. Bacteria-free allantoic fluids with HA activity were aliquoted and stored at −80 °C for further analysis, while the supernatant of homogenised embryonic tissues were used for the subsequent passage.

### Virus detection

Viral RNAs were extracted from HA-positive allantoic fluids and supernatants of homogenised tissue using TRIzol reagent (Gibco, Invitrogen) according to the manufacturer’s recommended procedure, and tested for IAV M-gene presence with VetMAX-Plus Multiplex One Step RT-PCR kit (Applied Biosystems, CA, United States [US]) and previously described primers and probes (Spackman et al. 2003). The reactions included 2 µL PCR-grade water, 6 µL 2× RT-PCR buffer, 0.5 µL RT enzyme mix, 0.5 µL of 10 pmol/µL of each primer, 0.15 µL of 5 pmol/µL of the probe and 3 µL of extracted RNA or positive control RNA or nuclease-free water as a no template control. The reactions were performed in an Applied Biosystems StepOnePlus RT-PCR system (Life Technologies, Carlsbad, CA, US) with the following cycling conditions: 1 cycle of 48 °C for 10 min; 1 cycle of 95 °C for 10 min; 40 cycles of amplification at 95 °C for 15 s and 53 °C for 45 s (data capture point).

### Ethical considerations

Ethical clearance for the study was obtained from the Animal Ethics Committee of the University of Pretoria (project number V010-17).

## Results

### Effect of mode of long-distance transport on embryonated ostrich egg viability

Transporting EOEs by air freight with a commercial courier resulted in a high proportion of broken eggs and poor viability of the remaining intact eggs (Table 1), presumably due to rough handling and fluctuating temperature through transportation process. In the first delivery batch, five of the 12 eggs were broken on arrival, and of the seven intact eggs, only one developed a viable embryo at the end of the 28-day incubation period. The second batch transported by a different courier arrived with all 12 eggs broken.

**TABLE 1:** Effect of mode of long-distance transport on embryonated ostrich egg viability.

Batch no.	Transport mode	Total no. eggs	Early embryonic deaths†		No. viable after incubation	
			*n/N*	%	*n/N*	%
1	Air freight courier service	12‡	0	-	1/7	14.0
1	Air freight courier service	12§	0	-	0	-
2	Road freight	24	5/8	62.5	0	-
3	Road freight	12	7/12	58.3	4/12	33.3
4	Road freight	12	5/12	41.7	1/12	8.3
5	Road freight	12	8/12	66.7	2/12	16.7
6	Air transport as hand luggage	6¶	1/6	16.7	5/6	83.3
7	Air transport as hand luggage	6¶	1/6	16.7	5/6	83.3
8	Air transport as hand luggage	6¶	1/6	16.7	5/6	83.3

no., number.

† , Infertility after 6 days incubation;

‡ , Five eggs broken in transit;

§ , Five eggs broken and seven damaged in transit;

¶ , Pre-incubated for 12–14 days prior to transport.

Transporting EOEs by road freight with the ostrich meat trucks substantially minimised handling and temperature fluctuations, and although a slight improvement on method one was noted, EOE viability was still poor. Between 41.7% and 66.7% of eggs arriving in four batches were confirmed as nonviable by day six of incubation, and of those that survived, only 8.3% to 33.3% were viable at 28 days.

Transporting EOEs as hand luggage by air yielded the best result. Of 18 eggs received in three batches of six eggs each, 15 (83%) were viable at day 28 of incubation and could be used in the subsequent experiments.

### Passage and isolation of influenza A viruses in embryonated ostrich eggs

Four successive passages were completed with the H5N2 LPAI virus in the 28-day old EOEs, whereas five passages were completed with the H7N1 LPAI virus and the allantoic fluids as well as the supernatants of homogenised embryonic tissues from each passage were tested for HA activity and by real-time RT-PCR (Table 2). The HA titres for H5N2-infected allantoic fluids increased with each passage from 2^5^ to 2^8^, whereas the titres for the corresponding tissue homogenates increased from 2^2^ to 2^4^. Higher HA titres in the allantoic fluids compared with the embryos are consistent with the site of replication in the egg of LPAI viruses. Low pathogenicity avian influenza viruses replicate in the cells of the choriallantoic membrane and virus particles are released during budding into the allantoic fluids (Rott et al. 1980). On HA, allantoic fluids containing the H5N2 LPAI virus showed complete haemagglutination, whereas embryonic tissue homogenates displayed incomplete haemagglutination (data not shown), consistent with the ALFs having higher virus titres than the embryonic tissues. Similar HA patterns were observed with the H7N1 LPAI virus, but the titres were generally higher ranging from 2^8^ to 2^10^ in the allantoic fluids. In the tissue homogenates, passage 1 had a higher HA titre of 2^5^, but from passage 2 onwards the values ranged from 2^3^ and 2^4^.

**TABLE 2:** Detection of influenza A viruses passaged in 28-day old embryonated ostrich eggs.

Virus	Passage no.	HA titre		Real-time RT-PCR Ct value	
		Allantoic fluid	Tissue homogenate	Allantoic fluid	Tissue homogenate
H5N2 LPAI	1	2^5^	2^2^	15.90	23.43
	2	2^7^	2^2^	14.40	19.93
	3	2^8^	2^3^	17.88	24.58
	4	2^8^	2^4^	16.53	23.64
H7N1 LPAI	1	2^8^	2^5^	12.61	15.43
	2	2^8^	2^3^	21.09	31.29
	3	2^10^	2^3^	11.84	21.17
	4	2^10^	2^4^	17.21	26.82
	5	2^10^	2^4^	16.91	18.23

HA, haemagglutinating activity; RT-PCR, real-time reverse transcription-polymerase chain reaction; Ct value, cycle threshold value; LPAI, pathogenic avian influenza; no., number.

Real-time PCR results confirmed that the haemagglutinating phenotype was caused by the presence of IAV in all samples. The average cycle threshold (Ct) values for H5N2 virus-infected allantoic fluids and embryonic tissue homogenates were 16.18 and 22.90 respectively, whereas those for the H7N1 virus were 15.90 and 22.70, respectively.

## Discussion

Embryonated ostrich eggs have the potential to be developed as *in ovo* passage models and for the selective propagation of IAVs that are ostrich-adapted; therefore, in this study the methods for transporting, incubating and inoculating these large eggs were described. Previously described challenges associated with the artificial incubation of ostrich eggs which include poor hatchability (± 45%), high rates of infertility (± 20%) and substantive in-shell deaths (± 30%), compared to that of other domestic poultry birds (Brand et al. 2014) were confirmed in this study. At the outset of the study, it was discovered that despite proper packaging to protect the eggs, the use of commercial air and road freight services either resulted in broken or damaged eggs or poor viability. The best method for long-distance transport of EOEs, although very expensive, was for a person to carry them as hand luggage by airline. It was also found that pre-incubating the eggs prior for 12 to 14 days prior to transport substantially improved the chances of incubating viable embryos to 28 days.

Designing experiments that involve the use of EOEs should take the timing of the ostrich breeding system into consideration. As opposed to specific pathogen-free chicken eggs, which may be obtained any time as required, ostrich eggs are only available during their breeding season, which coincides with increased photoperiod and may vary with altitude and latitude (Bertram 1979; Ipek & Sahan 2004; Smith et al. 1995). Breeding peaks around early spring in the northern hemisphere; whereas in the southern Africa, production spans June (mid-winter) to January (summer) (Lambrechts 2004).

The authors recently demonstrated, using 14-day ECEs, that passaging the same ostrich-origin H5N2 and H7N1 LPAI strains resulted in the appearance of the HPAI variants after 11 passages and seven passages for H5N2 and H7N1, respectively (Laleye & Abolnik 2020). The emergence of both H5 and H7 HPAI viruses from LPAI precursors in chickens is demonstrated in field outbreaks, *in vivo* and *in ovo* studies (Laleye & Abolnik 2020). In ostriches, H5N2 HPAI that emerged from LPAI precursors in field outbreaks are reported (Abolnik et al. 2016), but it is still unknown whether mutation to HPAI can occur with the H7 subtype. Field evidence suggests that H7 LPAI viruses are incapable of mutating to HPAI as H7N1 and/or H7N7 viruses have circulated in ostriches in South Africa and Italy on numerous occasions, sometimes for many months, without any evidence of conversion, and H7 HPAI has never been reported in ostriches in any country (Abolnik et al. 2009, 2016; Manvell et al. 2005). Future work could entail passaging the ostrich-origin H7N1 LPAI virus in 28-day EOEs to determine the risk of HPAI emergence.

## Conclusion

This study was the first to describe the methods for long-distance transport, incubation, inoculation and passage of IAVs in 28-day EOEs. The methods could be applied to isolate ostrich-adapted viruses and *in ovo* studies aimed at better understanding of the virus–host interaction in ostriches and the emergence of potentially zoonotic diseases.
